# Association of Original and Revised Pooled Cohort Equations With Cardiovascular and Cerebrovascular Mortality

**DOI:** 10.1002/cns.70737

**Published:** 2026-01-03

**Authors:** Chuanhao Lu, Min Li, Deyu Sun, Hongchen Zhang, Liang Li, Yang Yu, Juan Wang, Lele Cao, Zhen Hu, Xia Li

**Affiliations:** ^1^ Department of Neurosurgery, Xijing Hospital The Fourth Military Medical University China; ^2^ Department of Neurology, Xijing Hospital The Fourth Military Medical University China; ^3^ David Geffen School of Medicine University of California Los Angeles Los Angeles USA; ^4^ Institute for Health Informatics University of Minnesota Minneapolis USA

**Keywords:** cardiovascular mortality, cerebrovascular mortality, pooled cohort equations, vascular risk assessment

## Abstract

**Background:**

Cardiovascular (CVD) and cerebrovascular diseases (CeVD) are leading causes of mortality. The Pooled Cohort Equations (PCE) are widely used for ASCVD risk prediction, but face calibration concerns, particularly in diverse populations. The Revised Pooled Cohort Equations (RPCE) were developed to address these limitations. While prior research has evaluated these models for ASCVD, less is known about their performance for CVD and CeVD mortality outcomes.

**Objective:**

This study aimed to comprehensively compare the association of PCE and RPCE with CVD mortality and combined CVD and CeVD mortality, including subgroup analyses by race and gender, within a nationally representative US adult population.

**Methods:**

We analyzed 16,584 primary prevention participants (aged 40–79 years) from National Health and Nutrition Examination Survey (1999–2018), linked to National Death Index mortality data. We calculated 10‐year ASCVD risk using both PCE and RPCE. Bland–Altman plots assessed score agreement. Cox proportional hazards models evaluated the association of standardized PCE and RPCE scores with CVD and combined CVD and CeVD mortality, adjusting for confounders and stratifying by race and gender.

**Results:**

Both PCE and RPCE were significantly associated with CVD mortality (adjusted hazard ratio (HR) for PCE: 1.91 [95% CI: 1.82–2.01]; RPCE: 1.65 [95% CI: 1.59–1.72]) and combined CVD and CeVD mortality (adjusted HR for PCE: 1.91 [95% CI: 1.82–2.00]; RPCE: 1.65 [95% CI: 1.60–1.72]). Bland–Altman analyses revealed PCE consistently overestimated risk compared to RPCE, with differences increasing at higher risk levels. RPCE demonstrated improved calibration, especially in racially diverse populations, where PCE overestimation was more pronounced.

**Conclusion:**

Both PCE and RPCE are robust prognostic tools for vascular mortality. However, RPCE offers improved calibration, particularly in racially diverse populations, by providing more conservative yet comparably predictive risk estimates. These findings highlight the importance of selecting risk models tailored to target populations to optimize prevention and avoid potential overtreatment.

## Graphical abstract

This study compares the Pooled Cohort Equations (PCE) and Revised Pooled Cohort Equations (RPCE) in predicting cardiovascular and cerebrovascular mortality. Results show that while both models are robust, RPCE provides better calibration, especially in racially diverse populations, suggesting its use to optimize prevention strategies and avoid overtreatment.

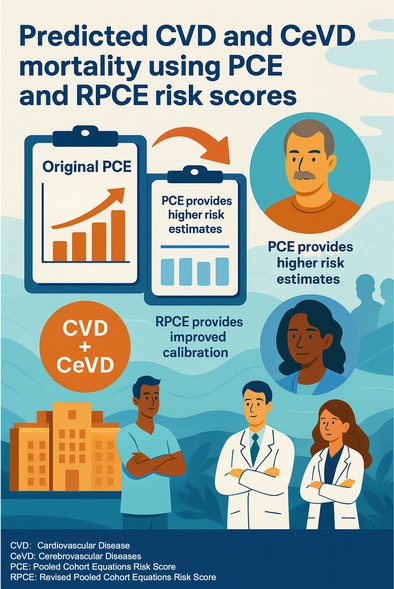

## 1 Introduction

Cardiovascular disease (CVD) and cerebrovascular disease (CeVD) continue to rank among the leading causes of morbidity and mortality worldwide, creating a significant burden in both healthcare and economic terms [1, 2, 3]. Therefore, effective risk assessment tools are crucial in both guiding clinical decision‐making and shaping public health policies [4, 5]. Among the various risk stratification models, the Pooled Cohort Equations (PCE), introduced by the American Heart Association (AHA) and the American College of Cardiology (ACC) in 2013, have gained prominence due to their ability to estimate a 10‐year risk of atherosclerotic cardiovascular disease (ASCVD) [6, 7]. This estimation uses easily accessible clinical and demographic factors—age, gender, race, lipid levels, blood pressure, history of hypertension treatment, diabetes, and smoking status—to derive an actionable percentage that clinicians can use when considering lifestyle modifications, additional testing, and interventions such as statin therapy [8, 9].

Recent evidence suggests that the PCE performs better than simpler cardiovascular health scores, including Life's Simple 7 (LS7) and Life's Essential 8 (LE8), for better risk discrimination of cardiovascular mortality [10]. Nonetheless, concerns persist regarding overestimation or underestimation of risk in specific subgroups. A large systematic review and meta‐analysis evaluated the performance of PCE across multiple cohorts and found that overestimation was particularly pronounced in high‐risk patients and discrimination performance was better in female patients [11]. As a result, in 2018, a Revised Pooled Cohort Equations (RPCE) model was proposed to improve calibration and applicability across diverse populations [12]. The PCE and RPCE both estimate 10‐year ASCVD risk using the same variables, but RPCE refits the model and recalibrates the baseline hazards [12]. Some studies have reported minimal improvements with the RPCE relative to the original PCE [13], whereas others have found notable benefits in particular populations, such as South Asians [9].

Another important gap in existing literature involves the singular focus of many prior studies on either ASCVD incidence or CVD mortality alone. There is established recognition that cerebrovascular disease shares similar risk factors with cardiovascular disease (e.g., hypertension, diabetes, dyslipidemia, and smoking) [14, 15, 16]. Consequently, evaluating both CVD‐specific mortality and a combined CVD and CeVD mortality outcome may better reflect real‐world clinical complexity and guide preventive care decisions [17, 18].

Given these considerations, this study aimed to compare the performance of the original PCE and the RPCE in associating with both CVD‐specific mortality and combined CVD and CeVD mortality. Leveraging data from the National Health and Nutrition Examination Survey (NHANES) from 1999 to 2018, we also conducted subgroup analyses to explore the differences by race and gender. We hope the findings could offer potentially valuable guidance to clinicians and policymakers choosing the most appropriate risk assessment tool for primary prevention across demographically heterogeneous populations.

## 2 Methods

### 2.1 Data Source and Ethical Statement

This study utilized data from NHANES (1999–2018), which is a nationally representative survey to study the health and nutritional status of the US population. Data collection includes household interviews, physical examinations, and laboratory testing, providing a comprehensive dataset for epidemiological research [19]. We used 1999 as the starting year because it marks the beginning of continuous collection of all components used for PCE and RPCE calculation. Mortality data used in this study were obtained by linking NHANES participants to the National Death Index (NDI), with follow‐up through December 31, 2019 [20]. The patients involved in the database have obtained ethical approval. Researchers can download relevant data for free for research and publish relevant articles. The study adheres to the principles of the Declaration of Helsinki.

### 2.2 Study Population

Consistent with the target population of the PCE and RPCE, we included participants aged 40–79 years when they got interviewed. We excluded individuals with a history of coronary artery disease, myocardial infarction, angina, stroke, or congestive heart failure to focus specifically on primary prevention. Participants who were pregnant or breastfeeding were excluded, given the unique physiologic and metabolic changes during pregnancy. Finally, individuals missing any key elements required for the PCE or RPCE calculations were excluded to ensure a complete dataset for both models.

### 2.3 Calculation of Risk Scores

Both the original PCE and RPCE models utilize the same set of variables, including age, gender, race, total cholesterol, high‐density lipoprotein (HDL) cholesterol, systolic blood pressure (SBP), antihypertensive treatment, diabetes status, and smoking status. Total cholesterol and HDL cholesterol levels can be directly obtained from the laboratory data. SBP was calculated as the average of all available blood pressure measurements. Antihypertensive treatment status was determined based on self‐reported use of hypertension medications during the survey period. Diabetes status was determined based on self‐reported responses to the question, “Has a doctor ever told you that you have diabetes?” and confirmed with laboratory results indicating fasting glucose levels ≥ 126 mg/dL. Smoking status was derived from self‐reported responses to the questions, “Do you currently smoke cigarettes?” and “Have you smoked at least 100 cigarettes in your life?” combined with laboratory measurements of cotinine levels > 10 ng/mL. Each participant's 10‐year PCE and RPCE risk scores were calculated using the R package “PooledCohort” [21].

The development of PCE and RPCE models took only non‐Hispanic White and non‐Hispanic Black individuals. Due to this limitation of the two models, participants of other races were assigned the non‐Hispanic White category for PCE or RPCE risk calculation.

### 2.4 Covariates

To assess the predictive power of PCE and RPCE for CVD mortality, and combined CVD and CeVD mortality, we adjusted for the following necessary confounders: race/ethnicity (Mexican American, non‐Hispanic Black, non‐Hispanic White, Other Hispanic, Other Race‐Including Multi‐Racial), body mass index (BMI), education level (less than high school, high school, some college, college, or above), poverty income ratio (PIR), activity level (yes, no, unable to do activity), and number of seeing a doctor or other healthcare professionals (none, 1, 2–3, ≥ 4). To avoid over‐adjustment, key components of the PCE and RPCE were not included as the covariates.

### 2.5 Study Outcomes

The outcomes of interest were CVD mortality and combined CVD and CeVD mortality. NHANES participants' mortality data were retrieved from the NHANES Linked Mortality File NDI. Each participant was assigned a mortality status (alive or deceased) and the underlying cause of death. Participants not linked to a death record were considered alive throughout the study period.

In the NDI mortality data, CVD mortality was defined using ICD‐10 codes I00–I09 (rheumatic heart diseases), I11 (hypertensive heart disease), I13 (hypertensive heart and renal disease), and I20‐I51 (ischemic and other heart diseases). CeVD mortality was defined using ICD‐10 codes I60‐I69, including subarachnoid hemorrhage, intracerebral hemorrhage, cerebral infarction, stroke not specified as hemorrhage or infarction, and other cerebrovascular diseases [22].

### 2.6 Statistical Analysis

To assess the agreement between PCE and RPCE risk scores, Bland–Altman analysis was employed. This method calculates the average of the two risk scores and the difference between them, enabling the identification of systematic biases or discrepancies across the range of risk score values [23, 24]. Bland–Altman plots were generated for the overall population and stratified by race (non‐Hispanic White, non‐Hispanic Black, and grouping all other groups into “All Other Races”) and gender. These stratifications allowed us to evaluate the potential subgroup‐specific differences in agreement and therefore provide insights into whether discrepancies between the two risk scores varied by race or gender.

Cox proportional hazards models were used to evaluate the associations between PCE and RPCE scores and the two outcomes of interest. To facilitate direct comparison and ensure consistent scaling across the models, both the PCE and RPCE scores were standardized prior to analysis. This standardization involved centering the scores around their mean and dividing by their standard deviation. Hazard ratios (HRs) and the corresponding 95% confidence intervals (CIs) were estimated for both unadjusted and adjusted models with the covariates. R packages “survival” and “survminer” were used to build the Cox models.

Subgroup analyses were conducted separately by race and gender to explore potential subgroup‐specific differences in the associations between risk scores and mortality outcomes. All analyses were conducted using R software (version 4.4.0). The Bland–Altman plots and Cox proportional hazards models comprehensively evaluated the agreement and associations of the PCE and RPCE risk scores across diverse subgroups.

## 3 Results

**FIGURE 1:**
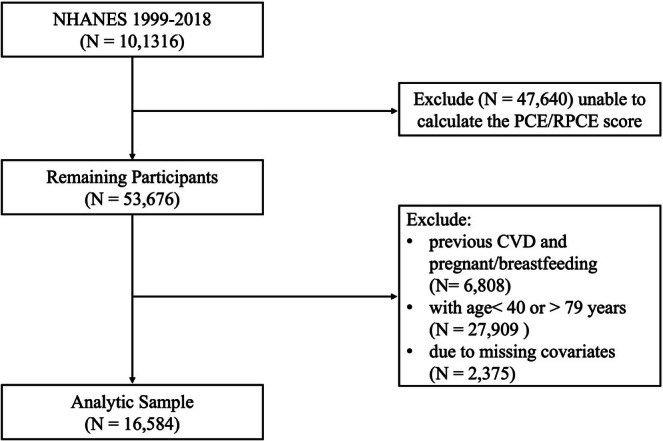
Study Population Flowchart.

In the beginning, we had the initial cohort of 101,316 participants. 47,640 individuals were excluded due to insufficient data to calculate their PCE or RPCE scores. Of the remaining 53,676 participants, we excluded 6808 individuals with self‐reported previous CVD. This brought the sample down to 46,868 participants. Subsequently, 27,909 individuals outside the age range of 40–79 years, which aligns with the age range of the PCE targeted population, were excluded. From the resulting 18,959 participants, 2375 were further excluded due to missing covariates needed for multivariable survival analyses. The final analytic sample consisted of 16,584 participants (Figure 1).

### 3.1 Baseline Characteristics

Table 1 outlines the baseline characteristics by PCE and RPCE risk categories. Participants were categorized into four risk groups according to their calculated 10‐year ASCVD risk score. Low risk was defined as a 10‐year risk of less than 5%, borderline risk ranged from 5% to less than 7.5%, intermediate risk ranged from 7.5% to less than 20%, and high risk was defined as a 10‐year risk of 20% or greater. These categories serve as practical benchmarks to inform preventive measures, like starting or adjusting statin therapy, and enable meaningful comparisons among different subgroups within the study population. Briefly, individuals classified as “High risk” by either model were generally older and more often male, which aligns with known epidemiologic patterns of cardiovascular risk. The largest racial group was non‐Hispanic White participants, but there was also a significant representation of individuals from various ethnic backgrounds. Additionally, the average BMI increased with higher risk categories, and there was an inverse relationship between educational attainment and risk category.

**TABLE 1:** Baseline characteristics of the study population across pce and rpce risk categories in the National Health And Nutrition Examination Survey (1999–2018).

	PCE low (*N* = 7634)	PCE borderline (*N* = 1984)	PCE intermediate (*N* = 4642)	PCE high (*N* = 2324)	RPCE low (*N*= 9557)	RPCE borderline (*N* = 1980)	RPCE intermediate (*N* = 3861)	RPCE high (*N* = 1186)
Age, mean (SD)	48.3 (6.3)	54.7 (7.7)	60.5 (8.2)	69.0 (7.4)	50.2 (7.8)	57.7 (9.1)	63.1 (8.8)	68.2 (7.6)
Gender, *n* (%)								
Female	5252 (68.8)	915 (46.1)	1897 (40.9)	833 (35.8)	6366 (66.6)	824 (41.6)	1378 (35.7)	329 (27.7)
Male	2382 (31.2)	1069 (53.9)	2745 (59.1)	1491 (64.2)	3191 (33.4)	1156 (58.4)	2483 (64.3)	857 (72.3)
Race, *n* (%)								
Mexican American	1512 (19.8)	325 (16.4)	771 (16.6)	390 (16.8)	1776 (18.6)	328 (16.6)	669 (17.3)	225 (19.0)
Non‐Hispanic White	3547 (46.5)	927 (46.7)	1951 (42.0)	993 (42.7)	4457 (46.6)	889 (44.9)	1601 (41.5)	471 (39.7)
Non‐Hispanic Black	1077 (14.1)	435 (21.9)	1157 (24.9)	643 (27.7)	1525 (16.0)	432 (21.8)	1006 (26.1)	349 (29.4)
Other Hispanic	691 (9.1)	163 (8.2)	417 (9.0)	160 (6.9)	861 (9.0)	167 (8.4)	317 (8.2)	86 (7.3)
Other Race/Multi‐Racial	807 (10.6)	134 (6.8)	346 (7.5)	138 (5.9)	938 (9.8)	164 (8.3)	268 (6.9)	55 (4.6)
BMI, mean (SD)	28.9 (6.7)	29.7 (6.5)	29.7 (6.3)	29.6 (6.0)	29.0 (6.7)	29.4 (6.4)	29.7 (6.2)	29.8 (6.0)
Education, *n* (%)								
Less than High School	1596 (20.9)	466 (23.5)	1333 (28.7)	801 (34.5)	2027 (21.2)	511 (25.8)	1206 (31.2)	452 (38.1)
High School	1512 (19.8)	463 (23.3)	1122 (24.2)	586 (25.2)	1945 (20.4)	498 (25.2)	940 (24.3)	300 (25.3)
Some College	2218 (29.1)	584 (29.4)	1275 (27.5)	546 (23.5)	2782 (29.1)	561 (28.3)	1013 (26.2)	267 (22.5)
College or above	2308 (30.2)	471 (23.7)	912 (19.6)	391 (16.8)	2803 (29.3)	410 (20.7)	702 (18.2)	167 (14.1)
Activity, *n* (%)								
Yes	2127 (27.9)	411 (20.7)	767 (16.5)	236 (10.2)	2532 (26.5)	360 (18.2)	542 (14.0)	107 (9.0)
No	5474 (71.7)	1559 (78.6)	3820 (82.3)	2049 (88.2)	6977 (73.0)	1594 (80.5)	3275 (84.8)	1056 (89.0)
Unable to do activity	33 (0.4)	14 (0.7)	55 (1.2)	39 (1.7)	48 (0.5)	26 (1.3)	44 (1.1)	23 (1.9)
Health Visit Number, *n* (%)								
None	1235 (16.2)	345 (17.4)	607 (13.1)	145 (6.2)	1526 (16.0)	304 (15.4)	418 (10.8)	84 (7.1)
1	1592 (20.9)	369 (18.6)	744 (16.0)	273 (11.7)	1929 (20.2)	347 (17.5)	571 (14.8)	131 (11.0)
2–3	2310 (30.3)	526 (26.5)	1407 (30.3)	695 (29.9)	2847 (29.8)	556 (28.1)	1202 (31.1)	333 (28.1)
> = 4	2497 (32.7)	744 (37.5)	1884 (40.6)	1211 (52.1)	3255 (34.1)	773 (39.0)	1670 (43.3)	638 (53.8)
PIR, mean (SD)	2.99 (1.68)	2.89 (1.69)	2.67 (1.63)	2.38 (1.50)	2.97 (1.68)	2.75 (1.63)	2.57 (1.59)	2.28 (1.50)
CVD mortality, *n*	45	36	181	292	84	57	228	185
CVD and CeVD mortality, *n*	52	44	224	358	99	71	282	226

We also conducted formal normality tests (Lilliefors and Anderson‐Darling) for BMI and PIR. As expected in a large sample (*N* = 16,584), these tests indicated statistically significant deviations from perfect normality. Because Cox proportional hazards models do not require normally distributed covariates, all continuous and categorical covariates were included as specified without transformation.

### 3.2 Comparison of PCE and RPCE Scores

**FIGURE 2:**
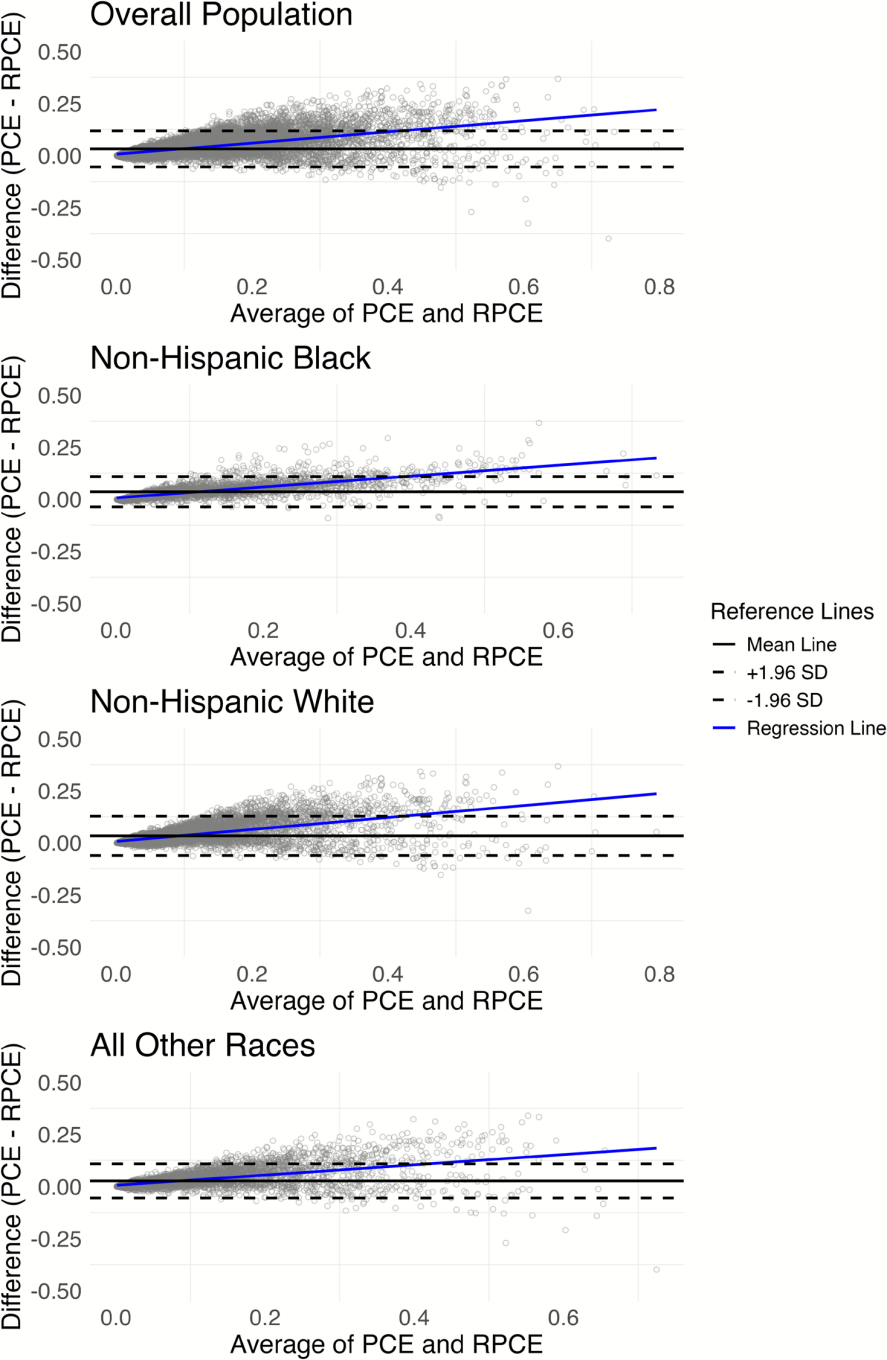
Bland–Altman Analysis of Agreement Between PCE and RPCE Risk Scores Across Racial Subgroups.

In Figure 2, the Bland–Altman plots visualize how well the original PCE and the RPCE risk scores agree across different racial subgroups. For the entire population in this study, we can find many data points outside the +1.96 Standard Deviation (SD) line, and the regression line shows an upward slope. As a result, PCE tends to systematically provide higher risk estimates than the RPCE, with this difference becoming larger and more variable at higher average risk levels. This pattern of increasing disagreement with a higher risk range is also observed in non‐Hispanic White individuals and those categorized as “All Other Races.” Among Non‐Hispanic Black participants, a similar trend of positive bias is observed, but relatively fewer data points are outside the ±1.96 line, which means the magnitude of disagreement may be somewhat less variable, particularly in the moderate to higher range of average risk scores. This suggests that the RPCE adjustments have helped align the scores more closely for this group compared to others.

**FIGURE 3:**
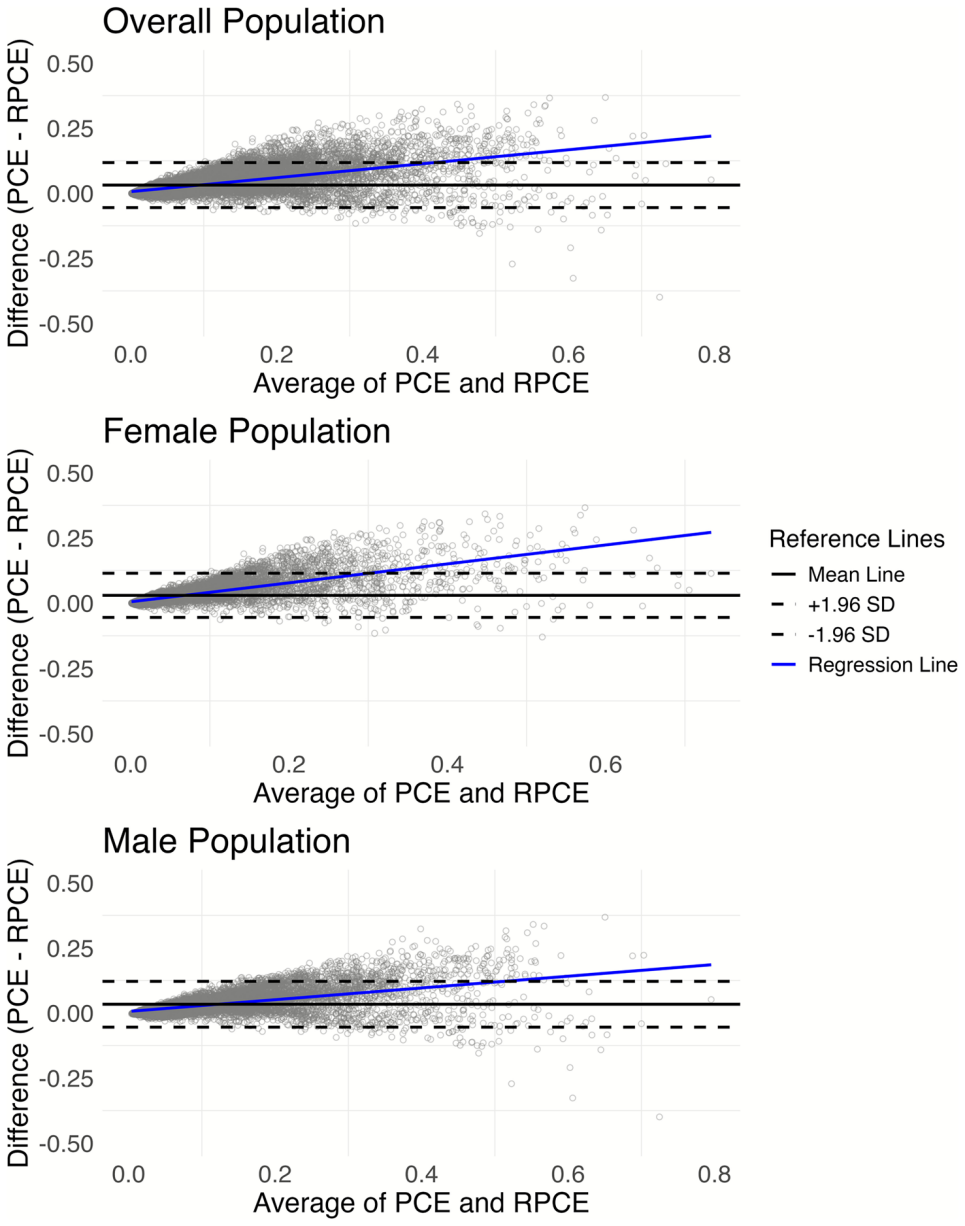
Bland–Altman Analysis of Agreement Between PCE and RPCE Risk Scores Stratified by Gender.

In Figure 3, the plots illustrate the agreement between PCE and RPCE risk scores for the overall population, females, and males. In both the female and male subgroups, as well as the overall population, there is a clear positive bias (i.e., upward sloping regression line), indicating that PCE scores are systematically higher than RPCE scores on average. Furthermore, the variability of the differences between the scores tends to increase as the average estimated risk rises for both genders. However, this trend appears more pronounced in females, where the spread of differences, particularly the degree to which PCE exceeds RPCE at higher risk levels, seems greater compared to males, suggesting a potentially larger disagreement between the two scores for women at higher risk estimates.

### 3.3 Association With CVD and Combined CVD and CeVD Mortality

Table 2 presents the associations between each standardized risk score and two outcomes of interest. For CVD mortality, in unadjusted Cox models, the PCE score was associated with a hazard ratio (HR) of 2.06 (95% CI: 1.97–2.16), while the RPCE score had an HR of 1.73 (95% CI: 1.67–1.80). After adjusting for demographic and behavioral factors, PCE remained a stronger predictor with an HR of 1.91 (95% CI: 1.82–2.01) compared to 1.65 (95% CI: 1.59–1.72) for RPCE. The ratio of HRs between PCE and RPCE was 1.16 (95% CI: 1.06–1.27), indicating a statistically significant improvement in predictive power with PCE over RPCE.

**TABLE 2:** Association between PCE and revised PCE with CVD mortality and combined CVD and CeVD mortality.

	Unadjusted model		Adjusted model	
	HR (95% CI)	Ratio of HR (95% CI)	HR (95% CI)	Ratio of HR (95% CI)
CVD mortality				
PCE risk score	2.06 (1.97–2.16)	1.19 (1.10–1.29)	1.91 (1.82–2.01)	1.16 (1.06–1.27)
RPCE risk score	1.73 (1.67–1.80)	Reference	1.65 (1.59–1.72)	Reference
Combined CVD and CeVD mortality				
PCE risk score	2.08 (1.99–2.18)	1.18 (1.09–1.28)	1.91 (1.82–2.00)	1.15 (1.06–1.25)
RPCE risk score	1.76 (1.70–1.83)	Reference	1.65 (1.60–1.72)	Reference

A similar pattern was observed for combined CVD and CeVD mortality. In unadjusted models, the PCE and RPCE scores had HRs of 2.08 (95% CI: 1.99–2.18) and 1.76 (95% CI: 1.70–1.83), respectively. After adjustment, the HRs were 1.91 (95% CI: 1.82–2.00) for PCE and 1.66 (95% CI: 1.60–1.72) for RPCE. The ratio of HRs was 1.15 (95% CI: 1.06–1.25), again suggesting that PCE is significantly more predictive than RPCE for vascular mortality encompassing both cardiac and cerebrovascular causes.

**FIGURE 4:**
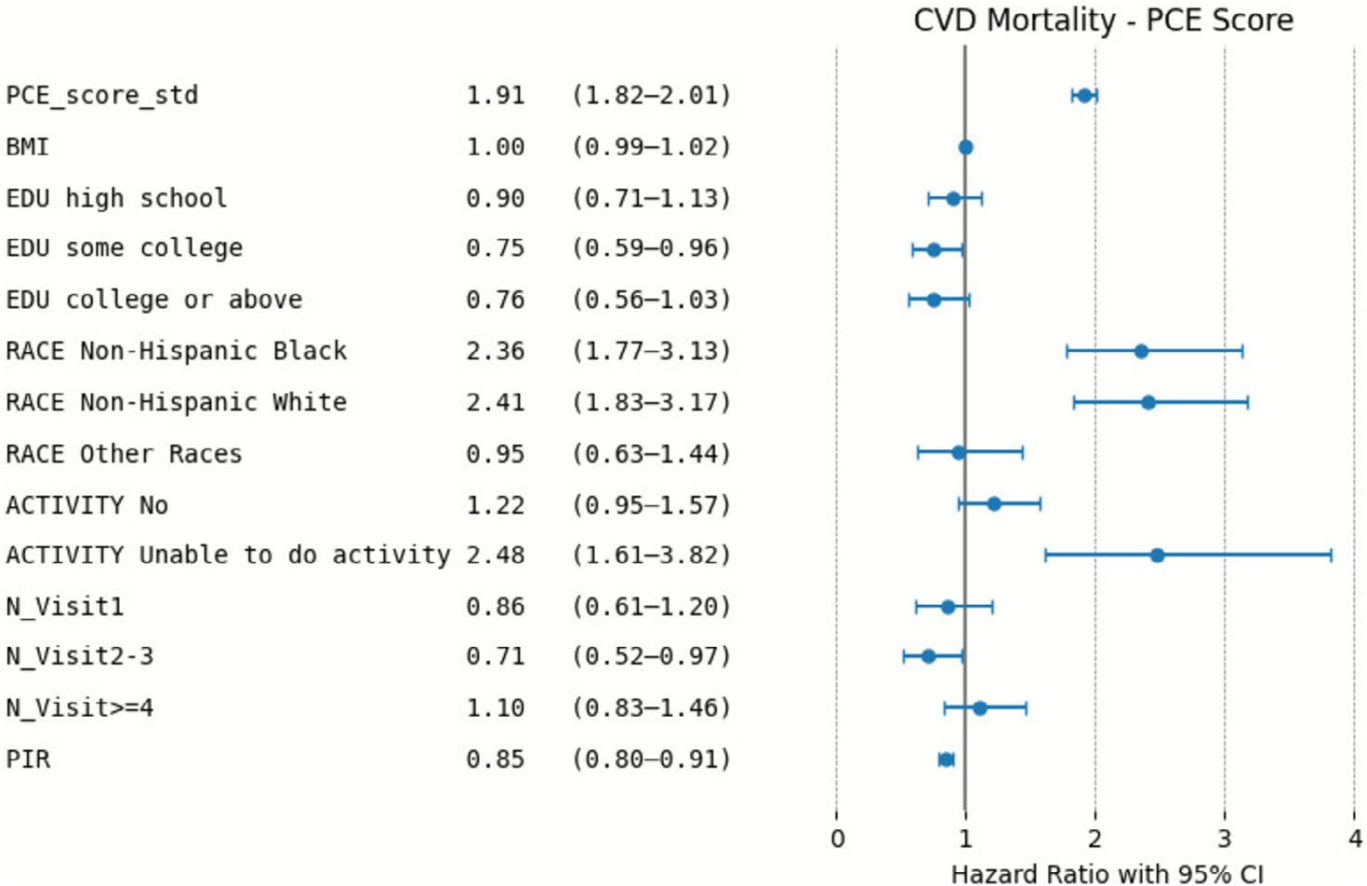
Risk Factors for CVD Mortality in the PCE Model: Adjusted Hazard Ratios.

**FIGURE 5:**
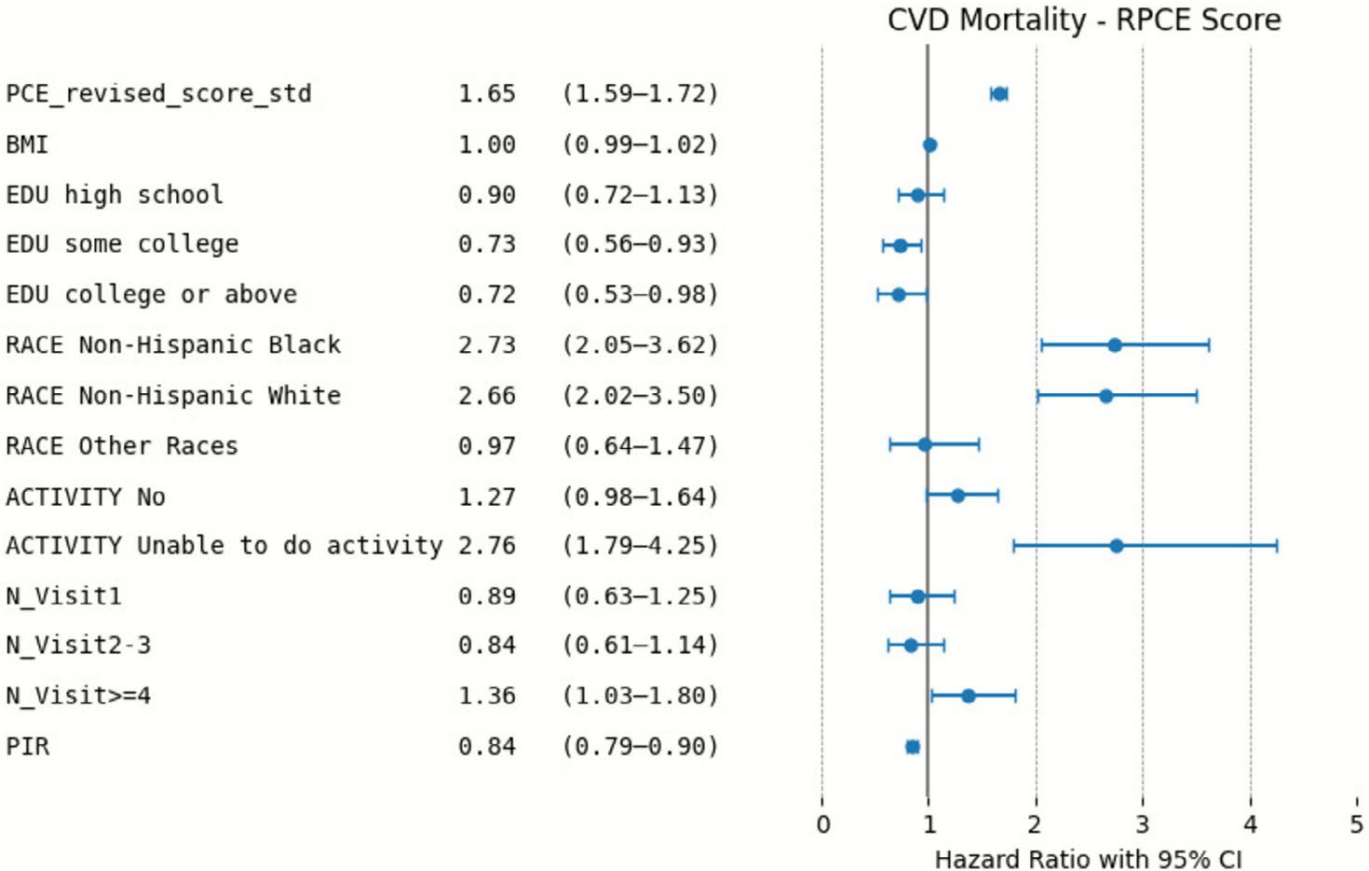
Risk Factors for CVD Mortality in the RPCE Model: Adjusted Hazard Ratios.

The forest plots in Figures 4 and 5 indicate again that both PCE and RPCE are significantly associated with CVD mortality. Factors such as non‐Hispanic Black and non‐Hispanic White race and inability to engage in physical activity were significantly associated with increased risk in both models, whereas higher PIR was protective as expected, as generally higher PIR indicates better socioeconomic status.

**FIGURE 6:**
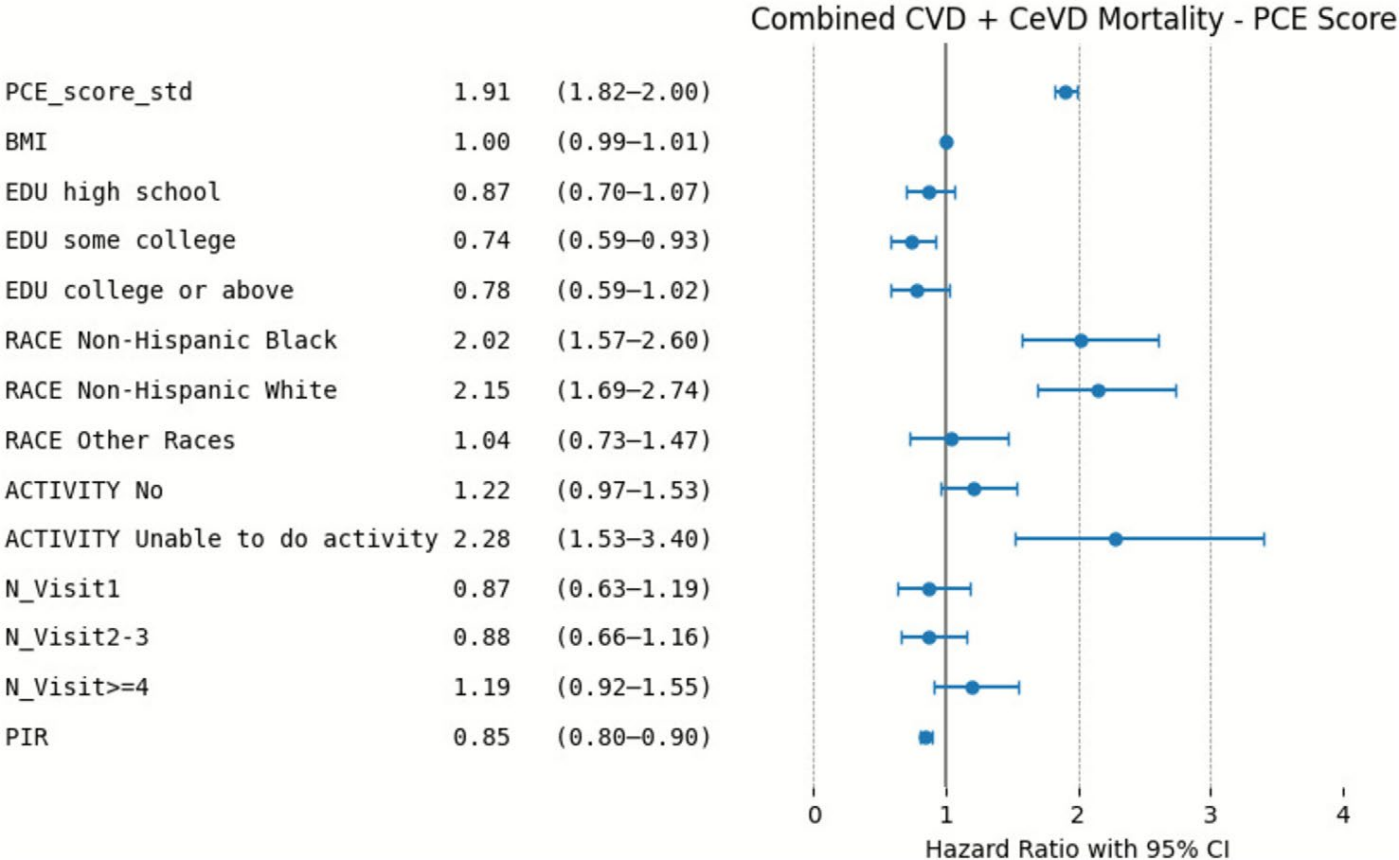
Risk Factors for combined CeVD and CVD Mortality in the PCE Model: Adjusted Hazard Ratios.

**FIGURE 7:**
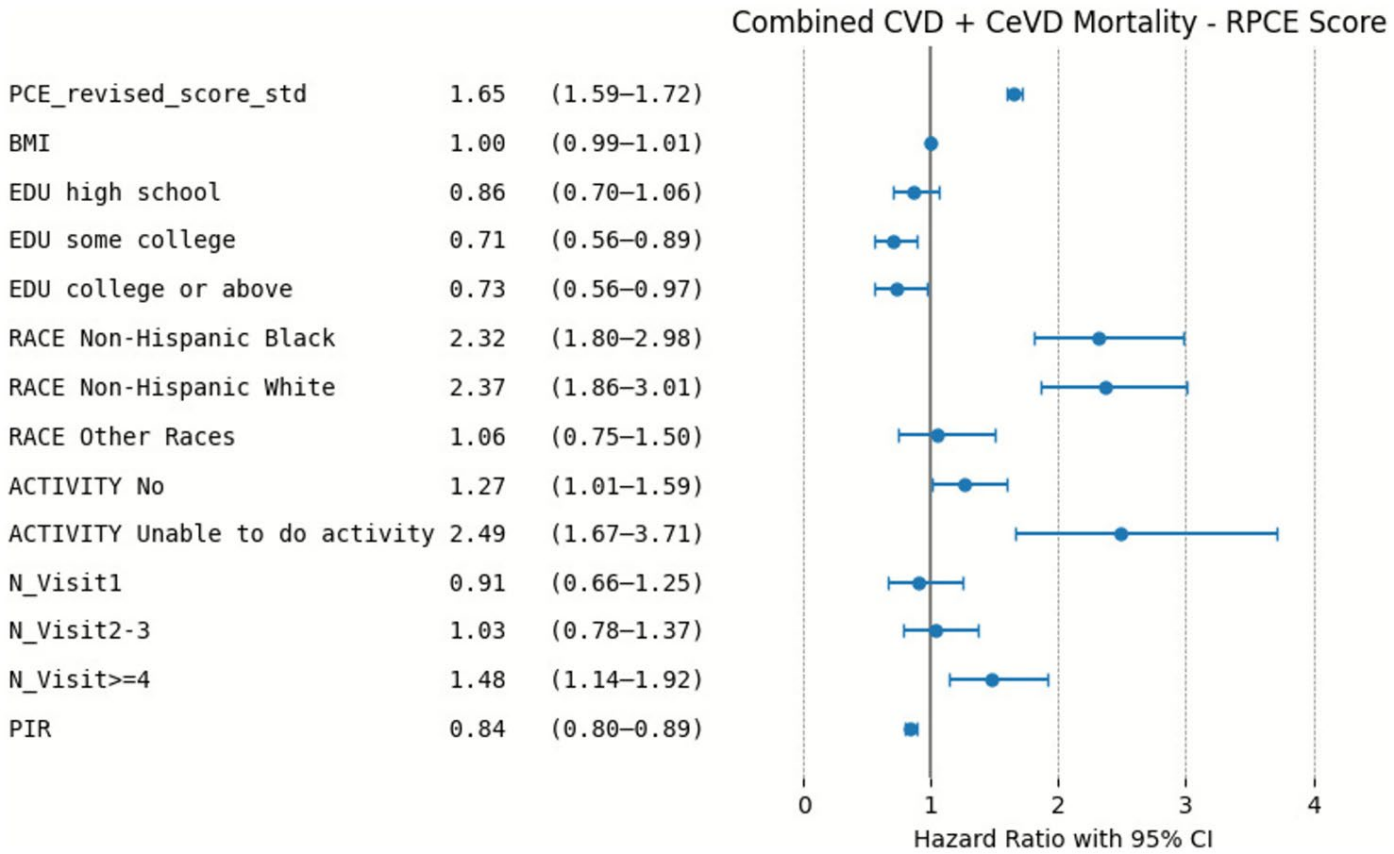
Risk Factors for combined CeVD and CVD Mortality in the RPCE Model: Adjusted Hazard Ratios.

Similar trends were observed in the combined outcome of CVD and CeVD mortality from Figures 6 and 7. Both PCE and RPCE provided significant predictions, with PCE again yielding higher risk estimates. RPCE scores offered more conservative risk estimates compared to PCE, which highlights its improved calibration. The results consistently show the increased risk associated with racial categories (particularly non‐Hispanic Black and White) and limited physical activity. Similarly, lower PIR was associated with higher risk of CVD and CeVD mortality.

### 3.4 Subgroup Analysis

In Table 3, the association between PCE and RPCE risk scores and CVD mortality demonstrated consistent trends across racial subgroups, with PCE scores generally yielding higher HRs than RPCE scores. Among non‐Hispanic Black participants, the unadjusted HR for PCE was 1.92 (95% CI: 1.76–2.09), slightly higher than the HR of 1.85 (95% CI: 1.70–2.00) for RPCE. After adjustment, the HRs were 1.52 (95% CI: 1.35–1.72) for PCE and 1.47 (95% CI: 1.32–1.64) for RPCE. The ratio of HRs (PCE vs. RPCE) remained close to 1 in both unadjusted (1.04; 95% CI: 0.88–1.23) and adjusted (1.03; 95% CI: 0.82–1.30) models, suggesting comparable predictive utility in this subgroup. In contrast, non‐Hispanic White participants exhibited higher HRs overall, with adjusted HRs of 1.55 (95% CI: 1.40–1.72) for PCE and 1.39 (95% CI: 1.29–1.50) for RPCE. The ratio of HRs for this group was slightly elevated in the unadjusted model (1.18; 95% CI: 1.06–1.32), but not statistically significant in the adjusted model (1.12; 95% CI: 0.94–1.33). Among “All Other Races,” the adjusted HRs were 1.31 (95% CI: 1.09–1.56) for PCE and 1.17 (95% CI: 1.02–1.34) for RPCE. A significant difference in the unadjusted ratio of HRs was observed (1.25; 95% CI: 1.03–1.51), though this difference was not significant in the adjusted model (1.11; 95% CI: 0.82–1.52).

**TABLE 3:** Association between PCE and revised PCE with CVD mortality and combined CVD and CeVD mortality for different racial groups.

	Unadjusted model		Adjusted model	
	HR (95% CI)	Ratio of HR (95% CI)	HR (95% CI)	Ratio of HR (95% CI)
CVD mortality—Non‐Hispanic Black				
PCE risk score	1.92 (1.76–2.09)	1.04 (0.88–1.23)	1.52 (1.35–1.72)	1.03 (0.82–1.30)
RPCE risk score	1.85 (1.70–2.00)	Reference	1.47 (1.32–1.64)	Reference
CVD mortality—Non‐Hispanic White				
PCE risk score	2.14 (2.02–2.28)	1.18 (1.06–1.32)	1.55 (1.40–1.72)	1.12 (0.94–1.33)
RPCE risk score	1.81 (1.73–1.90)	Reference	1.39 (1.29–1.50)	Reference
CVD mortality—All other Races				
PCE risk score	1.98 (1.78–2.21)	1.25 (1.03–1.51)	1.31 (1.09–1.56)	1.11 (0.81–1.52)
RPCE risk score	1.58 (1.46–1.72)	Reference	1.17 (1.02–1.34)	Reference
Combined CVD and CeVD mortality—Non‐Hispanic Black				
PCE risk score	1.91 (1.75–2.07)	1.03 (0.88–1.22)	1.50 (1.34–1.69)	1.03 (0.82–1.28)
RPCE risk score	1.83 (1.70–1.98)	Reference	1.46 (1.31–1.62)	Reference
Combined CVD and CeVD mortality—Non‐Hispanic White				
PCE risk score	2.13 (2.02–2.26)	1.17 (1.06–1.29)	1.53 (1.39–1.68)	1.09 (0.93–1.28)
RPCE risk score	1.82 (1.74–1.90)	Reference	1.39 (1.30–1.49)	Reference
Combined CVD and CeVD mortality—All other Races				
PCE risk score	1.98 (1.81–2.18)	1.24 (1.05–1.46)	1.30 (1.11–1.51)	1.09 (0.84–1.43)
RPCE risk score	1.59 (1.48–1.71)	Reference	1.18 (1.05–1.32)	Reference

For the combined outcome of CVD and CeVD mortality, similar patterns emerged. Among non‐Hispanic Black participants, the adjusted HRs were 1.50 (95% CI: 1.34–1.69) for PCE and 1.46 (95% CI: 1.31–1.62) for RPCE, with a corresponding HR ratio of 1.03 (95% CI: 0.82–1.28). For non‐Hispanic White participants, adjusted HRs were 1.53 (95% CI: 1.39–1.68) for PCE and 1.39 (95% CI: 1.30–1.49) for RPCE, with the ratio of HRs again modestly elevated in the unadjusted model (1.17; 95% CI: 1.06–1.29) but not significant in the adjusted model (1.09; 95% CI: 0.93–1.28). The “All Other Races” group showed adjusted HRs of 1.30 (95% CI: 1.11–1.52) for PCE and 1.18 (95% CI: 1.05–1.32) for RPCE, with an unadjusted HR ratio of 1.24 (95% CI: 1.05–1.46) and an adjusted ratio of 1.09 (95% CI: 0.84–1.43). These findings continue to support the observation that PCE may slightly overestimate risk compared to RPCE, especially in racially diverse populations, in line with RPCE's goal of improving model calibration.

Table 4 focuses on gender‐specific estimates of CVD and combined CVD and CeVD mortality. Across all outcomes, the PCE generated higher risk estimates compared to the RPCE in both unadjusted and adjusted models. Among males, the differences were statistically significant in both unadjusted and adjusted models. The ratio of HR for CVD mortality is 1.19 (95% CI: 1.05–1.36), and for combined CVD and CeVD mortality is 1.19 (95% CI: 1.06–1.34). However, among females, the differences between PCE and RPCE were often subtle and not statistically significant. These findings suggest that the RPCE provides more conservative risk estimates in males and better calibration in females, while maintaining comparable predictive performance overall.

**TABLE 4:** Association between PCE and revised PCE with CVD mortality and combined CVD and CeVD mortality for females and males.

	Unadjusted model		Adjusted model	
	HR (95% CI)	Ratio of HR (95% CI)	HR (95% CI)	Ratio of HR (95% CI)
CVD mortality—Female				
PCE risk score	2.09 (1.96–2.23)	1.09 (0.97–1.23)	1.94 (1.80–2.10)	1.09 (0.95–1.25)
RPCE risk score	1.90 (1.80–2.02)	Reference	1.78 (1.66–1.90)	Reference
CVD mortality—Male				
PCE risk score	2.01 (1.89–2.15)	1.22 (1.09–1.37)	1.89 (1.75–2.03)	1.19 (1.05–1.36)
RPCE risk score	1.64 (1.56–1.72)	Reference	1.57 (1.49–1.67)	Reference
Combined CVD and CeVD mortality—Female				
PCE risk score	2.07 (1.95–2.19)	1.09 (0.97–1.22)	1.92 (1.80–2.05)	1.09 (0.96–1.24)
RPCE risk score	1.90 (1.80–2.00)	Reference	1.77 (1.66–1.88)	Reference
Combined CVD and CeVD mortality—Male				
PCE risk score	2.03 (1.91–2.15)	1.22 (1.10–1.35)	1.89 (1.77–2.02)	1.19 (1.06–1.34)
RPCE risk score	1.66 (1.59–1.73)	Reference	1.59 (1.51–1.67)	Reference

## 4 Discussion

In this nationwide cohort of US adults, we observed significant associations between both the PCE and RPCE risk scores and vascular mortality outcomes (i.e., CVD mortality and the combined CVD and CeVD mortality), underscoring their utility in primary prevention. However, key differences between the models reveal important implications for clinical practice.

First, the RPCE demonstrated improved calibration in almost all racial groups from Table 3. We found that PCE consistently overestimated risk compared with RPCE, as reflected in its systematically higher risk estimates and the upward regression trends observed in Bland–Altman plots. Although PCE yielded significantly higher unadjusted HRs for non‐Hispanic White and “All Other Races” for both mortality outcomes, the adjusted HRs for both PCE and RPCE often demonstrated comparable predictive utility for both mortality outcomes in any racial subgroup. These findings indicate improved calibration, which aligns with the RPCE's goal of enhancing risk prediction for populations historically underrepresented in cardiovascular research.

Second, gender‐specific analyses revealed notable differences in risk prediction. For males, the PCE tended to yield significantly higher unadjusted and adjusted HRs compared to RPCE for both mortality outcomes. Adjusted HR ratio for CVD mortality is 1.19 (95% CI: 1.05–1.36) and for combined CVD and CeVD mortality is 1.19 (95% CI: 1.06–1.34), respectively. As the ratio is larger than 1, compared with RPCE, PCE scores are more strongly associated with mortality risk in men, while for females, the discrepancies between the two scores were less pronounced. These findings suggest that RPCE may offer more conservative risk estimation in males while maintaining comparable predictive utility in females. Importantly, the observed variability in agreement patterns between genders, as shown in the Bland–Altman plots, highlights the need for further calibration efforts to ensure that risk models perform equitably across gender subgroups.

From a clinical perspective, clinicians need to be careful about the choice between PCE and RPCE. While the PCE provides consistently higher absolute risk estimates for identifying individuals at risk, this may lead to overtreatment in populations where overestimation is more common—such as males or those with higher baseline risk. The RPCE, with its more conservative estimates, may help mitigate this risk, particularly in racially diverse or lower‐risk populations, while maintaining comparable predictive power in many subgroups. These findings emphasize the importance of shared decision‐making, where clinicians incorporate not only risk scores but also patient‐specific factors, such as comorbidities and preferences, when determining preventive strategies.

### 4.1 Strengths and Limitations

A major strength of this study lies in its comprehensive evaluation of both PCE and RPCE across a large, US nationally representative cohort with linked mortality data. The inclusion of combined CVD and CeVD mortality adds a novel dimension, providing insights into the broader implications of vascular risk prediction. Moreover, the use of standardized scores and subgroup analyses enhances the generalizability of our findings.

However, several limitations should be acknowledged. First, the reliance on self‐reported data for certain variables, such as smoking status, diabetes, and medication use, may introduce misclassification bias, though we leveraged both self‐reported data and lab results for smoking status and diabetes. Second, the relatively low event rate (~3%–4%) limits the precision of some estimates, particularly in smaller subgroups. Third, while NHANES is diverse, it may still underrepresent specific immigrant or marginalized populations, potentially limiting the applicability of findings to these groups. Finally, residual confounding by unmeasured factors, such as genetic predispositions or inflammatory markers, cannot be excluded.

### 4.2 Future Directions

Future research should prioritize the recalibration of these risk equations for less represented racial and ethnic groups. Additionally, longitudinal studies examining how temporal changes in risk factor profiles influence the utility of PCE and RPCE are warranted. Exploring real‐world implementation outcomes, such as clinician adoption and patient adherence to risk‐based recommendations, would further elucidate the practical utility of these models.

## 5 Conclusion

In conclusion, both the PCE and RPCE offer robust prognostic tools for predicting CVD mortality and combined CVD and CeVD mortality in the US adult population. While the PCE consistently produces higher risk estimates and presents stronger associations with the two mortality outcomes in this study, it also tends to overestimate risk particularly in males and individuals at higher baseline risk. Consistent with its original design purpose, the RPCE offers more conservative risk estimates and improved calibration, but it also tends to maintain comparable predictive utility. These findings highlight the importance of selecting risk models that align with the demographic and clinical characteristics of the target population. By integrating such insights into routine clinical practice, clinicians and policymakers can more effectively tailor preventive strategies and improve vascular health outcomes.

## Funding

National Natural Science Foundation of China (82371337, 82401531), Shaanxi Creative Talents Promotion Plan‐Technological Innovation Team (2022TD‐42), Shaanxi International Scientific and Technological Cooperation Project (2023‐GHZD‐21), Xijing Hospital Medical Staff Training Boost Special Plan (XJZT25CX34), Air Force Medical University Interdisciplinary Integration Project (2024JC002), Xijing Hospital Medical Staff Technical Skills Enhancement Program (2024XJSM14), Xijing Hospital Doctoral Dissertation Research Funding (2025BZ03), Shaanxi Health Scientific Research Innovation Capacity Enhancement Plan (2025TD‐17), Air Force Medical University 2025 Clinical Research Funding Program Project (2025LC2518).

## Disclosure

Artificial intelligence tools (specifically ChatGPT‐4o, developed by OpenAI) were used to assist with editing and polishing portions of this manuscript. The content of this manuscript, including the core research findings, analysis, and interpretation, is the original work of the authors and has been critically reviewed and validated for accuracy, quality, and integrity. Additionally, ChatGPT‐4o was used to generate the Graphical Abstract. The authors reviewed, refined and verified the content for accuracy.

## Conflicts of Interest

The authors declare no conflicts of interest.

## Data Availability

The data used are public de‐identified NHANES and linked mortality datasets from the US Centers for Disease Control and Prevention. They can be downloaded directly from the CDC website. The analytic code can be obtained from the corresponding author, Dr. Xia Li (Email: lixia_fmmu@163.com) on reasonable request.
